# Recent Advances in the Molecular Design and Applications of Multispecific Biotherapeutics

**DOI:** 10.3390/antib10020013

**Published:** 2021-03-30

**Authors:** Xiaotian Zhong, Aaron M. D’Antona

**Affiliations:** Department of BioMedicine Design, Medicinal Sciences, Pfizer Worldwide R&D, 610 Main Street, Cambridge, MA 02139, USA; aaron.dantona@pfizer.com

**Keywords:** multispecific biotherapeutics, immune cells engagers, antibody-drug conjugates, multispecific tetherbodies, biologic matchmakers, small-scaffold multispecific modalities

## Abstract

Recombinant protein-based biotherapeutics drugs have transformed clinical pipelines of the biopharmaceutical industry since the launch of recombinant insulin nearly four decades ago. These biologic drugs are structurally more complex than small molecules, and yet share a similar principle for rational drug discovery and development: That is to start with a pre-defined target and follow with the functional modulation with a therapeutic agent. Despite these tremendous successes, this “one target one drug” paradigm has been challenged by complex disease mechanisms that involve multiple pathways and demand new therapeutic routes. A rapidly evolving wave of multispecific biotherapeutics is coming into focus. These new therapeutic drugs are able to engage two or more protein targets via distinct binding interfaces with or without the chemical conjugation to large or small molecules. They possess the potential to not only address disease intricacy but also exploit new therapeutic mechanisms and assess undruggable targets for conventional monospecific biologics. This review focuses on the recent advances in molecular design and applications of major classes of multispecific biotherapeutics drugs, which include immune cells engagers, antibody-drug conjugates, multispecific tetherbodies, biologic matchmakers, and small-scaffold multispecific modalities. Challenges posed by the multispecific biotherapeutics drugs and their future outlooks are also discussed.

## 1. Introduction

Biopharmaceutical innovation has entered a new age of prosperity with record-breaking approvals for new molecular entities and biologics by the US Food and Drug Administration for the past few years [1]. Along with small-molecule entities, biotherapeutics drugs such as antibodies make up a significant portion of these approved new medicines. Novel therapeutic modalities such as RNA-based therapies, cell-based therapies, and gene-therapies are also available to patients who desperately need these medicines. The rise of this innovation wave builds on the rational drug design principle harnessing the power of genomics, proteomics, and metabolomics. The conventional mono-targeting drug model developed in the 1970s has worked exceptionally well on those “low-hanging fruit” targets [2,3], yet its effectiveness has been challenged by the growing complexity of yet-to-be addressed human diseases. Disappointing clinical studies and significant unmet medical needs remain abundant. As a response to these arduous tasks, the multispecific concept that targets two or more entities has been gradually picked up by the industry for the past decade. The multispecific biotherapeutics drug candidates have made up a significant portion of clinical and preclinical pipelines of large and small biopharmaceutical companies [4,5,6]. Developing multispecific drugs-based therapeutic agents have the potential not only to engage multiple therapeutic targets in disease pathways, but also to employ multiple modes of action and biological effectors. The power of antibody technology, the marriage between protein and chemistry (antibody drug conjugation), and the advancements of recombinant DNA technology, have put the biotherapeutics drugs into the forefront of the multispecific innovation wave.

The concept of artificially combining two distinct antigen binding paratopes targeting either the same or different antigens into one single molecule was first demonstrated by Nisonoff and Rivers sixty years ago [7]. A natural bispecific antibody formed by chain exchange of two IgG4 antibodies was later observed [8]. Current multispecific biotherapeutics can be roughly classified into two major categories, the non-obligate and the obligate multispecific [3,4,5,9,10,11]. Non-obligate multispecific biotherapeutics are exemplified by recombinant antibody-based molecules that bind two or more targets involved in independent signaling pathways (e.g., cytokines) thereby modulating these pathways simultaneously. Their efficacies could be similar to those of a mixture of separate antibodies, but the non-obligate multispecific biotherapeutics have the regulatory advantages of a single agent. In contrast, obligate multispecific biotherapeutics need the multiple binding specificities to be included within a single molecule to achieve expected efficacies. These molecules include immune-cell engagers (bringing immune cells to tumor cells to proximity for killing), antibody-drug conjugates (delivering small molecule drugs to target location via antibody), tetherbodies (enriching target molecules (cytokines or immunotoxin) into specific tissue/cellular locations), and biologic matchmaker drugs (close proximity to an action partner, e.g., emicizumab that brings substate Factor X (FX) to protease Factor IXa (FIXa) for cleavage).

For the obvious advantages in therapeutics application, taking the seemingly simple idea of multispecific-targeting into therapeutic reality has been challenging. The T-cell recruiting bispecific antibodies that killed tumor cells were first reported in 1985 [12,13]. Subsequently in the 1990s, a number of clinical trials were initiated testing the bispecific antibodies, yet with disappointing results the trials were discontinued [11]. Rapid and uncontrolled T-cell mediated cytokine release at very low dose of the drug hindered further attempts in development, along with issues in production and molecular instability. The final approval of blinatumomab for acute lymphocytic leukemia in 2014 marked the proof of principle of the T-cell engagement concept, though blinatumomab’s initial phase I trials (short-term intravenous infusion (IV), over 2–4 h at doses in the range of 0.75–13 µg/m^2^ up to three times per week) were terminated early due to instances of severe cytokine-release syndrome, neurotoxicity and lack of efficacy [5,14]. Only after the mode of administration was switched to long-term continuous IV infusion (for 4 or 8 weeks at doses in the range of 0.5–90 µg/m^2^ per day), impressive clinical results were finally achieved and sparked the evolution of the concept of bispecific T-cell engagers [4,5].

With a number of regulatory approvals for multispecific biotherapeutics drugs in recent years [3,4,5,6,15], this new innovative class of drugs have shown great therapeutic potential in a host of disease indications, such as cancer, rare diseases, inflammatory and autoimmune diseases, neurodegeneration, and infectious diseases. The topic of multispecific biotherapeutics represents a fast-growing field regarding formats, design strategies, and applications. A number of outstanding review articles have been written on this topic or related themes for the past few years [2,3,4,5,6,9,10,11,15,16,17,18,19,20,21,22,23,24,25,26,27,28,29,30,31,32,33]. This review summarizes comprehensively the recent advances in molecular design and applications of the multispecific biotherapeutics from the early stages of research discovery to the late stages of clinical and regulatory development. The article also focuses on the progress in therapeutic mechanisms of action, therapeutic strategies in drug development, and current landscapes of the major multispecific biotherapeutic drug classes. In addition, challenges and new opportunities are discussed from the perspectives of design and developability.

## 2. Major Classes of Multispecific Biotherapeutic Drugs

By definition, multispecific biotherapeutic drugs are protein-based therapeutic molecules that can engage multiple drug-target binding interfaces concurrently. The modalities of these biomolecules can be antibodies, different formats of antigen-binding fragments, small scaffold protein domains, peptides, enzymatic domains, protein receptors, chemical molecules, or oligonucleotides like anti-sense RNA or small interference RNA (siRNA). These biological entities are covalently linked together to form a single molecule to achieve multiple therapeutic modes of action. For the protein components of multispecific biotherapeutics, they are typically generated through recombinant DNA technology. For entities like chemical molecules, oligonucleotides, and some peptides, they are covalently conjugated to the protein moieties through in vitro manipulation.

Antigen-binding moieties are the dominant components for multispecific biotherapeutics as they are the main building blocks for further genetic fusion or chemical conjugation. The molecular designs for the antigen-binding moieties have been extensively reviewed [9,10], with around 100 different formats developed over the past two decades. These formats consist of various binding modules arranged in different domain repeats and fusion orders. As summarized in Table 1, the binding modules typically include fragments of antibody (Fabs), single-chain variable fragment (scFv), diabodies, single-domain antibodies (VHH from llama or camel, VNAR from shark), synthetic peptides, receptor domains, enzyme domains, CH2-based nanoantibodies or non-antibody scaffolds (Affibody, Fynomers, Monobodies, DARPins, Knottins, VLRs) [9,10,21,34,35]. The connections between these modules are either amino acid linkers encoded by DNA or chemical linkers synthesized in vitro.

One important criterion for multispecific polypeptides is the presence or absence of the antibody’s “fragment crystallizable region” (Fc) portion which plays a critical role in effector functions and FcRn-mediated recycling [36,37]. Based on the heterodimerization of Fc, multispecific molecules can be further classified into symmetric [9,10,38,39] or asymmetric architectures [4,30,40]. Symmetric multispecific biotherapeutics are homodimers with identical binding modules in each Fc fusion monomer, whereas asymmetric multispecific biotherapeutics are heterodimers with different binding modules in either of the Fc fusion monomers.

One unique problem posed for Fc-containing antibody-like multispecific biotherapeutics is the “chain-pairing” issue (detailed discussions are seen in several excellent reviews [4,9,10,30]), due to their need for the correct assembly of two antibody-like heavy chain (HC) polypeptides, and two or more antibody-like light chain (LC) polypeptides into the final multispecific molecules. A number of technologies have been developed to overcome this “chain-pairing” problem. As summarized in Table 2, various genetic alternations in the polypeptides, which introduce specific protein-protein interaction fits through shapes, charges or domain swaps between protein chains, have been proven successful in producing correctly assembled multispecific antibodies.

**Table 1:** Building blocks of multispecific biotherapeutics.

Binding Modules	Binding Modules	Molecular Weight	Molecular Properties	References
Fragments of antibody (Fabs)	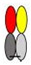	~50 kDa	Medium half-life/less aggregated	[9,10]
Single-chain variable fragment (scFv)	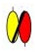	~25 kDa	Short half-life/less aggregated	[9,10]
Diabodies (Db)	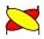	~25 kDa	Short half-life/less aggregated	[9,10]
Single-domain antibodies (VHH from llama or camel, VNAR from shark)	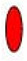	~15 kDa	Short half-life/less aggregated	[9,10]
Synthetic peptides	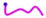	~3 kDa	Extremely short half-life/less aggregated	[41,42,43,44]
TCR domains	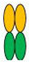	~50 kDa	Medium half-life/less aggregated	[45,46,47,48]
Enzyme domains	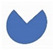	~50 kDa	Medium half-life/less aggregated	[49]
Small scaffolds (Affibody, Fynomers, Monobodies, DARPins, Knottins, VLRs, Nanoantibodies)	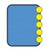	~5–12 kDa	Short half-life/less aggregated	[21,50,51,52,53,54,55,56]
Chemical molecules	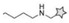	~3 kDa	Short half-life/aggregated	[6,17]
Oligonucleotides	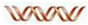	~10 kDa	Short half-life/less aggregated	[19,57]

**Table 2:** Strategies and mutations to ensure cognate light and heavy chain assembly in bispecific antibodies with the numbering of the European Union amino acid sequence (All are in human IgG1 unless mentioned otherwise).

Technology Name	Mutations in First Chain	Mutations in Second Chain	References
Knobs-into-holes (Genentech)	HC1: S354C, T366W	HC2: Y349C, T366S, L368A, Y407V	[58,59]
Electrostatic steering (Amgen)	HC1:K409D, K392D	HC2: D399K, E356K	[60]
Electrostatic steering (Pfizer)	IgG1 HC1: D221E, P228E, L368E IgG2 HC1: C223E, P228E, L368E	IgG1 HC2: D221R, P228R, K409R IgG2 HC2: C223R, E225R, P228R, K409R	[61]
Electrostatic Steering (Merus)	HC1: L351D, L368E	HC2: L351K, T366K	[62]
Fab-arm exchange (Genmab)	HC1: K409R	HC2: F405L	[63,64]
SEED (EMD Serono)	HC1: IgG/IgA chimera	HC2: IgG/IgA chimera	[65]
LUZ-Y (Genentech)	HC1: cleavable leucine zipper	HC2: cleavable leucine zipper	[66]
HA-TF (Xencor)	HC1: S364H, F405A	HC2: Y349T, T394F	[67]
EW-RVT (EW-RVTs-s) (Ajou University)	HC1: K360E, K409W (Y349C)	HC2: Q347R, D399V, F405T (S354C)	[68,69]
ZW1 (VYAV-VLLW) (Zymeworks)	HC1: T350V, L351Y, F405A, Y407V	HC2: T350V, T366L, K392L, T394W	[70]
DMA-RRVV (SYMV-GDQA) (UNC/Eli Lily)	HC1:K360D, D399M, Y407A (Y349S, K370Y, T366M, K409V)	HC2: E345R, Q347R, T366V, K409V (E356G, E357D, S364Q, Y407A)	[71]
Protein A affinity (Regeneron)	HC1: H435R	None	[72]
Protein A and Protein G Avidity (Glenmark)	HC1: IgG3Fc, N82aS	HC2: M428G/N434A/K213V	[73]
CrossMab (Roche)	HC1:CL-VH	LC1:CH1-VL	[74]
Fab-Interface engineering (Eli Lily)	HC1: Q39K, R62E, H172A, F174G LC1: D1R, Q38D, L135Y, S176W	HC2: Q39Y LC2: Q38R	[75]
Fab-Interface electrostatic steering (Amgen)	HC1: Q39K, Q105K, S183D LC1: Q38D, A43D, S176K	HC2: Q39D, Q105D, S183K LC2: Q38K, A43K, S176D	[76]
Κλ-bodies (Novimmune SA)	LC1:κ	LC2:λ	[77]
Common Light Chain (Genentech & Merck KGaA)	Shared LC	Shared LC	[78,79]
Tetravalent IgG-like Charged CR3 mutant (Biomunex)	Mab1 CH1: T192E	Mab1 CL: N137K, S114A	[80]
Tetravalent IgG-like Hydrophobicity-polarity swap MUT4 mutant (Biomunex)	Mab1 CH1: L143Q, S188V	Mab1 CL: V133T, S176V	[80]
Fabs-in-Tandem (FIT-Ig) (EpimAb)	Long chain: VL_A_-CL-VH_B_-CH1-CH2-CH3	Short Chain A: VH_A_-CH1, Short Chain B:VL_B_-CL	[81]
DuetMab (AstraZeneca)	CH1: F126C	CL: S121C	[82,83]
BEAT (Glenmark)	HC1-CH3: Residues from TCR α interface	HC2-CH3: Residues from TCR β interface	[84]
TCR CαCβ (Eli Lily)	HC1-CH1: TCR Cα	LC1:CL: TCR Cβ	[47]
WuXiBody (WuXi Biologics)	HC1-CH1: TCR Cβ	LC1-CL: TCR Cα	[48]

Non-protein entities utilize small molecules and oligonucleotides to greatly broaden the mechanism of action and design versatilities of the multispecific biotherapeutics [6,16,17,18,19]. They enable specific binding to intracellular drug targets such as those involved in replication and division of cells and DNAs. These non-protein moieties are covalently linked to protein backbones (typically antibodies) that target specific surface antigens in cells and tissues. Upon the arrival to either the extracellular space or inside cells through endocytosis, the conjugated chemical molecules or oligonucleotides are released to exert therapeutic effects.

**Figure 1:**
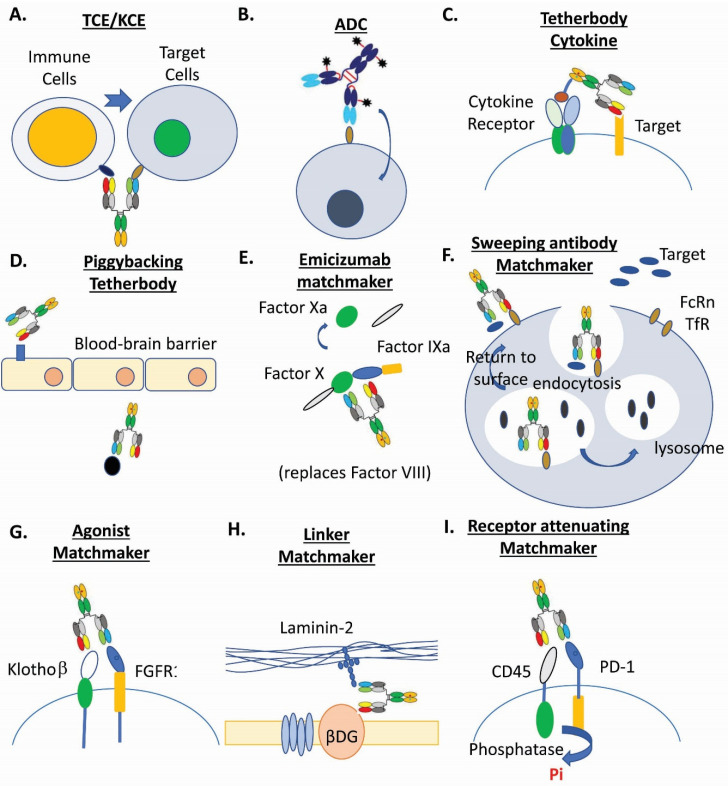
Examples of multispecific biotherapeutics drugs. (**A**). T-cell and NK-cell engagers (TCE/KCE) [4,5,11]. (**B**). Antibody-drug conjugates (ADC) [3,6,15,17,18,31]. (**C**). Tetherbody cytokine [95]. (**D**). Piggybacking tetherbody [96,97,98]. (**E**). Emicizumab matchmaker [99,100]. (**F**). Sweeping antibody matchmaker [3,101]. (**G**). Agonist matchmaker [102,103]. (**H**). Linker matchmaker [104]. (**I**). Receptor attenuating matchmaker [105].

The current generation of multispecific innovative drugs have ridden a wave of promising clinical and regulatory successes. As of January 2021, thirteen multispecific molecules have been commercialized (Table 3). Among them, there are three approved bispecific antibodies (catumaxomab (Removab^TM^), blinatumomab (Blincyto^®^), emicizumab (Hemlibra^®^)), one approved antibody binding fragment fusion with immunotoxin (Lumoxiti^TM^), and nine approved antibody-drug conjugates (gemtuzumab ozogamicin (Mylotarg^®^), brentuximab vedotin (Adcetris^®^), ado-trastuzumab emtansine (Kadcyla^®^), inotuzumab ozogamicin (Besponsa^®^), enfortumab vedotin (Padcev^TM^), fam-trastuzumab deruxtecan-nxki (Enhertu^®^), polatuzumab vedotin-piiq (Polivy^TM^), belantamab mafodotin-blmf (Blenrep^TM^), sacituzumab govitecan (Trodelvy^TM^)). These successes, along with nearly 200 multispecific biotherapeutic candidates in the clinical pipelines [3,4,5,6,10,16,17,18,19,20,21,22,23,24,25,26,27,28], indicate that multispecific biotherapeutics are promising molecules. However, discovering and developing them into an approved product is challenging and requires extensive efforts in engineering and development. The following sections summarize the recent advances of the major classes of multispecific biotherapeutics drugs (Figure 1) in designs and applications.

### 2.1. Immune-Cell Engagers

Directing immune cells to target tumor cells represents one major group of mechanism of action for the multispecific biotherapeutics. These so-called immune-cell engagers are the quintessential example of the obligate multispecific biotherapeutics which take advantage of the presence of different binding specificities within a single polypeptide molecule to achieve a new function. They are designed to form an artificial cytolytic synapse by bringing immune cells, such as cytotoxic T-cells and natural killer (NK) cells, into close proximity with tumor cells [4,5,11,23]. Subsequent release of granzymes and perforin from activated T or NK cells into tumor cells results in tumor cell death. In the meantime, activated NK cells can also evoke target cell caspase activation via TNF-related apoptosis-inducing ligand and Fas ligand. Versatile factors can be secreted by NK cells for modulating functions of other immune cells.

The formats for the immune-cell engagers are generally classified into IgG-like molecules and non-IgG-like molecules [4,5,9,10], each with advantages and disadvantages. IgG-like molecules retain the Fc component that may endow effector functions and FcRn-mediated recycling for longer serum half-lives, whereas large sizes of these molecules could hinder their tissue penetration. To generate multispecific IgG-like engagers that interact with both immune cells and tumor cells, a number of antibody formats have been reported. Quadroma relies on the fusion of two distinct hybridomas [85], through which catumaxomab is produced in a rat/mouse quadroma cell line. Engineering chain-pairing scaffolds with “Knobs-into-holes” [58,59] have been widely used [86,87,88], whereas electrostatic steering strategies [60,61] have been shown with successful applications [89,90]. Dual-variable domains Ig (DVD-Ig) [91,92] (formed by the fusion of the variable domains of two antibodies in tandem), IgG-scFv [9,10] (produced by fusing scFv or Fv to the termini of antibody chains), κλ bodies [77] (sharing same heavy chain with two different light chains (κ or λ) with highly selective affinity resins for purification), trivalent format [93], and various tetravalent formats [80,81,94] have been utilized for the design of new immune-cell engagers.

Non-IgG-like molecules do not contain Fc, but rather have different antigen binding fragments. They can become dimers, trimers, or tetramers depending on linker length and fragment sequences. A number of non-IgG-like formats have been reported: scFv-based molecules (exemplified by blinatumomab with the fusion protein containing light-chain variable domain (VL) and heavy-chain variable domain (VH), or tandem scFvs), diabody format (single-chain diabody, tandem diabodies, exemplified by dual-affinity retargeting molecules (DART)), and nanobodies from llamas, camels, or shark. The smaller size of the non-IgG-like formats offers advantages like enhancing tissue penetration, ease of manufacturing, and accessing sterically hindered epitopes. Their drawbacks are short half-lives and poor target-site retention. Besides the improvement with constant administration in the case of blinatumomab, a number of strategies have been put in place for serum half-life extension [106]. Attachment of highly flexible and hydrophilic molecules such as polyethylene glycol, carbohydrates, *N*-(2-hydroxypropyl) methacrylamide, dextran, and amino acid polymer XTEN [107] can increase the hydrodynamic volume. Fusions with human serum albumin (HSA) or albumin-binding domain like scFv to HSA can increase half-life significantly [108,109]. Additionally, multimerization can increase sizes and binding valency of the molecules [10,29].

#### 2.1.1. Multispecific T-Cell Engagers

Tumor-specific T cell clones play an important role in the immune surveillance against cancer [110], yet their amounts are limited in tumors. Down regulation and loss of the major histocompatibility complex (MHC) molecules in cancer cells often contribute to their escape of recognition and killing by tumor-infiltrating T-cells [23,111]. Bispecific T-cell engagers (bsTCEs) tackle this challenge by redirecting non-tumor specific T cells through the binding to CD3ε of T-cell receptors (TCRs) by one arm and to tumor cells via the binding to tumor-associated antigen (TAA) by the other arm. This process includes the formation of a transient cytolytic synapse and the subsequent activation and proliferation of T cells that steers to the lysis of tumor cells [23,112,113]. bsTCEs bypass MHC restriction and result in T cell activation independent of the epitope specificity of the TCRs. The majority of bsTCEs are developed for the treatment of hematological malignancies, while a significant number of bsTCEs at clinical stages target solid tumor antigens [4].

For T-cell redirection, CD3ε has been the most common and advanced target [4], although alternative T cell targets such as αβTCR and T cell surface glycoprotein CD5 [80] have also been explored in preclinical models with promising results. For the design of bsTCEs, extensive studies have shown that the affinity and valency of anti-CD3ε could significantly affect the biodistribution, safety, and efficacy of the therapeutics. Although bsTCE with high affinity (K_D_ < 1 nM) to CD3ε exhibited outstanding in vitro efficacy, those with a lower affinity (K_D_ of 50–200 nM) minimized CD3-mediated plasma clearance or trapping of spleen and lymph nodes, showed efficient tumor distribution in vivo [114,115,116,117]. The vast majority of bsTCEs in development contain monovalent of anti-CD3 arm, which is expected to prevent CD3 crosslinking resulting in unintended excess cytokine release. A few clinical-stage bsTCEs do contain bivalent anti-CD3 binding domain, though it is not clear if CD3 engagement is indeed bivalent [4,118,119]. They showed strict tumor-antigen-dependent T cell activation in vitro.

For tumor cell targeting, high-affinity binding is quite desirable. Besides an intrinsic dissociation constant of the parent molecules, the affinity can be further influenced by the architecture of the bsTCEe either positively (avidity effect) or negatively (steric hindrance by the attachment of additional units) [4,5,16]. Strohl and Naso have proposed that the affinity to TAA should be at least ten-fold higher than that to CD3ε [23]. This would allow bsTCE to be distributed to the tumor and coat TAA-positive tumor cells while attracting T-cells [23]. High-affinity bsTCEs (30–300 pM) could induce T-cell killing at low antigen density levels of 10–150 molecule per cell [120]. It has been proposed that a certain threshold of target expression is required for effective killing by bsTCEs, which varies depending on a specific antigen [4]. Tumor-specific neoantigen (peptides of intracellular proteins such as TP53 and RAS family are loaded onto classical human leukocyte antigen class I molecules on the cell surface) can be recognized by bsTCEs even presented with extremely low densities, and activated T-cells for cancer cell killing in mice [121,122].

Size and structure of target antigens, epitope location, and orientation of binding interaction also contribute to the efficient killing of tumor cells by bsTCE [86,123,124,125]. The dimensions of the target antigens have been shown to be critical for efficient T-cell synapse formation [125]. The normal space in the cytolytic synapses has been estimated to be about 13 nm [126]. Experimentally extended distances between the cell membranes decreased TCR activation [126], and the exclusion of large inhibitory molecule CD45 from the synapses is a key TCR triggering feature [127,128]. Target antigens with large size might not fit into the immunologic synapse formed by bsTCE. Rational epitope selection for bsTCE design are therefore critical for potent killing of tumor cells by overcoming the steric hinderance. In this regard, a small target size and a membrane-proximal binding epitope seem preferable [125]. Moreover, the preferred epitope location and binding orientation can be driven by the molecular formats and configuration of bsTCEs [39,129].

Antitumor activity of bsTCE can be limited by the development of treatment resistance. One potential mechanism is the downregulation of target antigen on tumor cells, such as CD19-relapses found in blinatumomab-treated patients [130]. Trispecific-TCE (TriTCE) has been therefore developed to target dual antigens to potentially overcome such resistance [131]. Treatment resistance of bsTCE also involves a state of T-cell non-responsiveness, i.e., T cell anergy or exhaustion. Protein molecule combinations of CD28-costimulatory receptor-TAA bispecifics with CD3-bsTCEs, providing sustained T-cell proliferation, markedly improved antitumor activities in a variety of tumor models [132]. Similarly, a TriTCE which engaged with CD3, CD28 and TAA in a single molecule was recently developed to enhance both T cell activation and tumor targeting with outstanding preclinical efficacy [133,134]. To overcome the treatment inhibition by checkpoint pathways, clinical trials are currently underway for combining bsTCE (Blincyto^®^, anti-CD19 BiTE^®^) with anti-PD-1, anti-PD-L1 or anti-CTLA4 antibodies [23]. Protein molecule combinations of CD28-TCEs with a PD-1 blocker resulted in an enhanced antitumor efficacy in preclinical models [135]. A novel TriTCE (called CiTE) that simultaneously targets a TAA, CD3, and PD-1/PD-L1 has shown efficacy in preclinical cancer models for prostate and epithelial tumors [136,137].

#### 2.1.2. Multispecific NK-Cell Engagers

Despite the successes of blinatumomab and catumaxomab, therapies targeting T-cells have toxicity issues, such as deadly cytokine release syndrome (CRS) and neurotoxicity. Balancing the full therapeutic potential with limited toxicity demands further investigations. NK cells have been known to mediate anti-tumor responses without prior sensitization to tumor antigen since they were first identified in the 1970s [138]. Unlike T cells, which recognize tumor antigens in the context of MHCs through TCR, NK cells utilize germline encoded receptors to recognize ligands present on cells as a result of viral infection or tumor transformation [11,139,140]. Due to this frontline role of innate immunity in tumor killing and good safety profiles, redirecting NK cells has long been an attractive strategy [11,139,140]. The mechanisms of NK cells in eliminating cancer cells are through T-cell-like perforin and granzymes induced cell lysis, TNF-related apoptosis, and immune cells-modulating factor secretion [140,141].

Bispecific NK cell-engagers (bsKCEs) was in the second wave of clinical studies following those of bsTCEs in the 1990s. Anti-CD16 was the arm directing NK cells with another arm against CD30 in a F(ab’)2 format for end-stage Hodgkin’s disease [11,142,143]. The studies showed some encouraging clinical results but were discontinued due to low production yield and high immunogenicity. Interests in bsKCE have been renewed in recent years as progress in protein engineering has overcome its early limitations. Formats of bsKCE include joining scFvs against an NK-cell receptor (CD16, NKp46, or NKG2D) and a TAA (CD19, CD20, CD30, CD33, CD133, CD38, GPC3, and EPCAM) [11,119,139,140,144,145,146,147,148]. A tetravalent bispecific tandem diabody against CD16A and CD30, discovered by Affimed, efficiently triggered NK cells-mediated lysis of CD30^+^ lymphoma [119,148].

While CD16 and NKG2D are among the first to be used for engaging NK cells, NKp46 is becoming a hot target since NKp46 is more sustainably expressed in tumor-infiltrating NK cells, whereas CD16 and NKG2D are frequently down-regulated [149]. NKp46 is also more NK-cell specific, while NKG2D is widely expressed in T-cells which has a higher risk of inducing T-cell-associated toxicities, such as CRS [150]. In addition, NKp46-mediated NK activation has been shown to prevent metastasis and help reshape tumor microenvironment [151]. Symmetric IgG-like bsKCEs against NKp46 and tumor antigen GPC3 are being developed by Cytovia [80,147]. TriKCEs targeting more TAAs or co-engaging more NK cell activating receptors should exhibit better efficacy, as Innate Pharma’s TriKCEs targeting both NKp46 and CD16 have shown with potent in vivo tumor killing activity that appeared superior to those of therapeutic antibodies [149]. To overcome the poor persistence limitation of NK cells in vivo, a cytokine IL15 key for NK cell development and proliferation was fused to anti-CD16 and CD33 bsKCE [152]. The resulting TriKCE elicited superior NK cytotoxicity and NK cell persistence in a xenograft tumor model.

In addition, the tumor microenvironment is known to suppress NK cells’ function and result in tumor escape and disease progression [11,139,140]. scFvs targeting checkpoint receptors like KIRs, TIGIT, NKG2A, or PD-1 have been proposed to be included in TriKCE constructs to further drive NK-mediated anti-tumor responses [139]. Interestingly, Affimed is also combining KCEs with NK cells, by combining its AFM13 or AFM24 with autologous NK cells (SNK-01) from NKMax America [153]. Cytovia, the first company to possess both induced-Pluripotent Stem cell (iPSC) Chimeric Antigen Receptor (CAR)-NK and NK engager platform technologies, also plans to combine its allogeneic universal iPSC-NK cells with its NKp46 bsKCEs [147].

### 2.2. Antibody Drug Conjugates

Antibody drug conjugates (ADCs) (Table 4) are an important class of multispecific biotherapeutics that combine two or more binding activities into one entity [3,6,15,17,18,31]. They are composed of chemical compounds (cytotoxic drugs or nucleotide acids) covalently linked to an antibody component that binds to a specific target. The selective binding to the target brings the conjugated chemical components into specific cells or tissues where they can exert their therapeutic effects. This should significantly limit the undesirable side effects of cytotoxic drugs or oligonucleotides. ADCs are often considered as prodrugs that are supposed to be nontoxic in circulation. A key attribute for an ADC is the average number of payloads (conjugated drugs) per antibody or the so-called drug-to-antibody ratio (DAR). The DAR can affect an ADCs’ potency and toxicity as well as plasma stability, biophysical properties, and pharmacokinetics.

Similar to that of immune-cell engagers, the successful development of an ADC depends on the selection of an appropriate target antigen for the antibody component. Besides high tumor-association to minimize on-target toxicity of ADCs, the antigens engaged by ADCs need to be endocytosed upon antibody binding. High copy numbers of antigens’ surface expression in tumor cells (>10^5^/cell, [154]) is crucial for the efficacy of ADCs because as more surface antigens bind to the ADC, then more chemical drugs can be delivered into the tumor cells [6,17]. Since most TAAs also express in normal tissues at lower levels, bispecific ADCs targeting two TAAs should increase tumor cell specificity. Such molecules have been recently shown to increase ADCs’ internalization and reduces tumor resistance in preclinical models [155].

For designing an effective ADC, selection criteria for cytotoxic payloads should include strong cell toxicity, preservation of potency after conjugation release, acceptable aqueous solubility, stability in aqueous formulation and physiological conditions, as well as synthetic feasibility under good manufacturing practice [6,15,17,18,31]. Most cytotoxic drugs are hydrophobic and tend to induce antibody aggregation which cause fast clearance and acute host responses [17,156]. The current major categories for cytotoxic payloads are microtubule inhibitors and DNA-damaging agents. Antimitotic agents cause cell death by interfering with the ability of mitotic spindles to segregate chromosomes and alter cellular cytoskeletal architecture [154,157]. The two most widely used antimitotic agents for ADCs are based on auristatins and maytansinoids [154,157]. For DNA-damaging agents, they are DNA binding agents that attach to the double-helix and promote DNA strand alkylation, scission or crosslinking [154,157]. The representative examples for this agent class are calicheamicins, duocarmycins, camptothecins, anthracyclines, and pyrrolobenzodiazepines. Other mechanisms of action for ADCs’ cytotoxic payloads include Topoisomerase I inhibitor-based payload, RNA splicing inhibition using natural product-derived thailanstatin, and RNA polymerase I inhibition using α-amanitin derivatives [18]. Besides small molecule payloads, synthetic oligonucleotide therapeutics (antisense oligonucleotides and siRNA) [19,57,158] have been conjugated into the antibody backbone showing efficacy in vivo and in vitro [159,160,161]. These payloads can target the RNAs encoding those intracellular proteins that are inaccessible and un-druggable by antibodies alone.

Linkers that connect payloads to antibodies are another critical feature of ADCs [6,15,17,18,31]. They must be stable in circulation and able to release the drugs in active form in the targeted location. The intrinsic features of the linkers and the payloads determine the physicochemical properties of ADC metabolites in nearby cells. As a result, the linkers induce a “bystander effect” to kill neighboring cells [6,15,17,18,31]. They can be classified into non-cleavable (like the one in trastuzumab emtansine) and cleavable (like the one in brentuximab vedotin). Non-cleavable linkers remain attached to the drugs after proteolytic degradation of the antibody in lysosome, which with linkers and an amino acid or short peptide are released into the cytosol to exert their activities [162]. The most common example of a non-cleavable linker is the thioether linker. For cleavable linkers, they exploit the differences between bloodstream and cytoplasm of targeted tissues. They can be cleaved by certain specific proteases found in lysosomes, or by low pH (acidic environment), or by a reducing environment (high glutathione concentrations in cytoplasm) to release free drugs [163]. Peptide linkers, hydrazone, and disulfide are a typical type of cleavable linkers.

Conjugation strategy is another important aspect for ADCs. Linker-payload moieties are typically conjugated to antibodies via lysine or cysteine residues. Ideally, the conjugation process should maintain the integrity of antibodies and the activity of drugs, as well as produce homogeneous batches that remain stable during the storage and lyophilization process [17]. Because the IgG scaffold has over 80 lysine residues, about 20 of which are highly solvent-accessible [164], the conjugation process results in highly heterogenous mixtures. For cysteine conjugation, four interchain disulfides in one IgG1 also lead to a heterogeneous ADC [165]. More homogeneous ADCs can be produced through site-specific conjugation via genetic engineering [6,15,17,18,31,166,167]. Engineered cysteine substitution at light chain and heavy chain positions provides reactive thiols and allows site-specific drug conjugation [168,169,170,171]. Unnatural amino acids capable of bio-orthogonal chemical conjugation can be introduced to antibodies through amber codon suppression [172]. Transglutaminase couple glutamine residues side-chain with linker-payloads through amide bonds [173,174]. Sortase catalyzed the ligation of the LPXTG tag at the C-terminus of a polypeptide with the N-terminal oligoglycine [175]. Glycotransferases can be engineered to transfer modified monosaccharides that enable bio-orthogonal chemical conjugation to a linker-payload [176]. Formylglycine-generating enzyme can covert an engineered cysteine residue in a specific peptide sequences such as CXPXR to produce an aldehyde tag for linker conjugation [177]. Most recently, engineered methionine residues have been utilized for efficient site-specific drug conjugation [178,179], and ADP-ribosyl cyclases enable a facile approach for generating site-specific ADXs CD38 fusion proteins [49].

### 2.3. Tetherbodies

Tetherbodies are a class of multispecific biotherapeutics drugs that utilize one binding interface to engage a docking protein for action enrichment to a particular location, with another binding interface to engage an effector protein. Regarding the mechanism of action, tetherbodies are similar to ADC, but its modalities are all protein-based and produced by recombinant DNAs [3].

The typical examples for tetherbodies are antibody-immunotoxin fusion (Lumoxiti^TM^) or antibody-cytokine fusion [95]. Immuotoxin or Cytokines (such as IL-2) have adverse effects when administered systemically. When fused to an antibody that binds to a TAA, immunotoxin or cytokines like IL-2, tetherbodies can be enriched and interact with receptors at those tumor sites. This interaction can greatly limit the toxicities of the resulting tetherbodies. Another example for tetherbodies is the “piggyback” or “hijacking” approach [3,4]. Because blood-brain barrier (BBB) restricts access of large molecules to the brain parenchyma, multispecific biotherapeutics with one antibody arm against plasma membrane receptors, e.g., transferrin receptor (TfR) that transcytose through brain endothelial cells, have been explored for efficient brain entry [96,97,98,180,181]. The efficacies of these tetherbodies have been demonstrated in animal models [97,181], yet clinical successes remain to be seen. Identifying novel receptors that are brain endothelial cell-specific represents a new opportunity. A similar piggyback mechanism for tetherbodies has been used for the indication of infectious diseases. By targeting persistent factor Psl and the needle tip protein PcrV of *Pseudomonas aeruginosa* [182], the resulting bsAb can increase internalization and localization in acidic vacuoles for neutrophil killing. In addition, by utilizing one arm targeting an extracellular exposed epitope in the filovirus glycoprotein (GP) of Ebola virus to gain access to endosome during viral uptake [183], a “Trojan horse” bispecific antibody used the other arm against a cryptic receptor binding site of GP or the corresponding Niemann-Pick C1 intracellular receptor to block virus entry to cytoplasm.

One novel subclass of tetherbody-like multispecific biotherapeutics drugs is the condition-activated tetherbody that can enrich the active drugs in a particular location through the design of conditional activation. The conditional activation mechanisms take advantage of special conditions in tumor microenvironment such as increased levels of specific proteases [184], ATP [185,186], and others [20]. Because the presence of tumor antigen in healthy tissues can cause on-target toxicity by ADCs or bsTCEs, Probodies^TM^ (CytomX) with a protease-cleavable peptide linker and a masking peptide can presumably remain inert until proteolytically activated locally in disease tissues [184]. A novel class of ADC called Probody-drug conjugates has shown the potential to protect normal tissues expressing target antigens [187]. The probody for EGFRxCD3 bsTCE has reported a 60-fold increase in dose tolerance compared to the unmasked construct [20,25]. A similar conditional activated TCE called ProTIA (protease-triggered immune activator) has been developed by Amunix and its AMX-168 is an anti-EpCAM BiTE-TCE fused to a masking XTEN polymer by a protease-cleavable linker [25].

### 2.4. Biologic Matchmaker Drugs

Biologic matchmaker drugs are a relatively new class of multispecific biotherapeutics that utilize one binding interface to engage an action partner, with another binding interface to engage a target [3]. They bring the drug targets in close proximity to an action partner for efficient biological activation or inactivation.

The best known example of a biologic matchmaker drug is emicizumab that replaces the scaffold function of factor VIII for generating activated FX [99,100]. Through hetero-binding arms, this bispecific antibody brings the substrate FX to the protease enzyme FIXa. A similar biologic matchmaker is a molecular linker that connects laminin-211 and the dystroglycan beta-subunit [104]. A novel bispecific antibody targeting both laminin and dystroglycan proteins has been developed to ameliorate sarcolemmal fragility for improving muscle function [104].

Biomimetics for signaling molecules are another type of biologic matchmaker. Bispecific agonistic antibodies that engage with FGFR1 with one arm and Klothoβ with another arm [102,103] have been developed to mimic fibroblast growth factor 21 for its signaling action. Most recently, bispecific biologic matchmakers have been constructed to recruit promiscuous cell-surface phosphatase CD45 to attenuate signaling of kinase-activated cell surface receptors such as PD-1 and SIRPα through dephosphorylation [105].

There are also other novel formats for biologic matchmaker drugs in early stage preclinical research. One example is the multispecific biotherapeutics that utilized the mechanism similar to heterobifunctional proteolysis-targeting chimeric molecules (PROTACs) [188,189,190]. Through the PROTACs’ first ligand to the target and the second ligand to ubiquitin ligase, PROTACs induce ubiquitylation of the target followed by proteolytic degradation in the proteasome [3]. A similar PROTACs-like multispecific biotherapeutics has been produced by Kanner et al. [191]. This PROTACs-like matchmaker consisted of a llama single-chain antibody fused to the catalytic domain of E3 ligase which can increase targeted ubiquitination of ion channels and consequentially diminish the surface expression of these membrane proteins. Interestingly, an opposite application has also been recently reported by the same research team. A multispecific deubiquitinase, in which a deubiquitinase was fused to a nanobody, deubiquitinated trafficking-deficient ion channels and consequentially rescued their functional surface expression [192]. Similarly, a multispecific matchmaker, named sweeping antibody, utilizes a lysosomal degradation system without taking advantage of proteasome activity [3]. This multispecific matchmaker binds to either FcRn or a surface antigen (i.e., TfR) in one arm at both neutral and acidic pH, with another arm interacting with the target only at neutral pH [3,101]. When the multispecific is endocytosed to endosome, the target is then released and proceeds to lysosome for degradation.

### 2.5. Small-Scaffold Multispecific Modalities

As an alternative to antibodies, various scaffold proteins have been developed and utilized for multispecific targeting [21,34]. They are Fynomers, Monobodies, Affibodies, DARPins, Knottins, Anticalins, synthetic peptides, CH2-based nanoantibodies, and others. These small-scaffolds have the advantages of small size, good stability, good tissue penetration, and reasonably high binding affinity, serving as robust building blocks for multispecific biotherapeutics.

Fynomers are among those best-characterized small-scaffolds that can be engineered to bind to targets of choice with antibody-like affinity and specificity [193]. They are small stable globular proteins (~7 kDa) derived from the SH3 domain of human Fyn kinase without disulfide bonds [193]. A bispecific antibody (FynomAb) was generated by fusing a HER2-specific Fynomer to either the N or C-terminus of a heavy or light chain of pertuzumab [55], showing a superior anti-tumor activity. COVA322 is a clinical-stage bispecific TNF/IL17-A inhibitor with IL-17A-specfic Fynomer fused to adalimumab [194], displaying good efficacy for inflammatory diseases.

Monobodies’ scaffold folding is similar to immunoglobulin domains but does not rely on the formation of an intradomain disulfide bond [195,196]. They are small scaffolds (~10 kDa) derived from fibronectin type II domain (FN3) [195,197]. Centyrins are structurally similar to Monobodies, but from the FN3 domain of Tenascin C [198]. A single loop and the face of a β-sheet form their diverse binding surfaces [50,196,199]. A bispecific Monobody-ubiquitin ligase fusion was generated for target degradation [200]. Two anti-EGFR Centyrin cytotoxic conjugates showed good IC50 values of around 0.2 nM [201].

Affibodies can be functionally produced both by peptide synthesis and by recombinant bacterial expression [202]. They are small scaffolds (~7 kDa) derived from a 58-amino-acid alpha-helical Z-domain of Staphylococcus protein-A [51,203,204]. They can display high affinity to a variety of targets such as HER2, selected from combinatorial libraries [202]. Anti-HER2 cytotoxic Affibody conjugate was generated and showed inhibition activity on tumor growth [205]. Recently, a trispecific affibody fusion protein consisting of anti-amyloid beta, a scFv against TfR and an albumin binding domain, was shown to increase uptake into the cerebrospinal fluid 24 h after injection [206].

Designed ankyrin repeat proteins (DARPins) are small stable scaffolds (~15–18 kDa) derived from Akyrin repeats which utilize their repetitive structural units to form an extended binding surface [207]. Anti-EpCAM DARPin toxin fusion [208] and anti-HER2 toxin fusion [209] have shown strong anti-tumor activity in vivo. Recently, a novel bispecific anti-EGFR DARPin fused with the inhibitory prodomain of ADAM17 was shown to decrease cell proliferation and migration of EGFR-dependent cancer cells [210]. MP0250, a trispecific DARPin in a Phase I/II study for advanced solid tumors, can bind to VEGF-A and hepatocyte growth factor as well as human serum albumin [22,211]. Anti-EpCAM DARPin fused to arginine-rich Low Molecular Weight Protamine protein was used for efficient siRNA delivery [212].

Knottins (~3–6 kDa) are cysteine knots with 30–50 amino acid residues exhibiting excellent stability [54]. Integrin-targeting knottin peptide-drug-conjugates showed potent inhibition on tumor cell proliferation [213]. Variable lymphocyte receptors (VLRs) are utilized by jawless vertebrates for adaptive immune recognition [53], and contain leucine-rich repeats found in mammalian innate immune receptors. They have been developed as binding scaffolds to therapeutic targets like IL6 [214]. Anticalins are derived from lipocalins and exhibit a structurally conserved αβ-barrel with an attached α-helix supporting hypervariable loops to bind protein molecules [215]. PRS-343, an agonistic anticalin targeting costimulatory receptor 4–1BB with an antibody against human HERs, are currently in clinical-stage development [216].

Human Ig constant CH2 domains (CH2 of IgG, IgA and IgD, and CH3 of IgE and IgM) have been proposed and utilized as a novel small-binding scaffold [35]. They have been coined nanoantibodies as they can be diversified by random mutagenesis and contain binding sites with stability and effector functions. Bispecific nanoantibodies have been engineered to interact with FcRn and HIV-1 neutralizing epitope [56].

Specific peptides can also be used for the fusion to an IgG to generate multispecific molecules either through genetic fusion [41,42] or chemical conjugation with a catalytic antibody called CovX-Bodies [43,217,218]. The small size of peptides provide advantages of epitope access and design versatility [44]. Zybody technology enables up to five target-specificities [42], but their degradation susceptibility requires special chemical modifications [41,218]. Peptide-conjugates utilize peptides such as those derived from natural receptors [219] or bicyclic peptides have shown better tumor penetration [18]. Peptide-antibody fusions have been recently generated against Middle East Respiratory Syndrome Coronavirus [220].

## 3. Challenges and Future Direction

The clinical and regulatory successes as well as the stockpiling in clinical and preclinical pipelines have made multispecific biotherapeutics drugs the most rapidly expanding group of therapeutic molecules for the past decade. Because of the inherent nature of engaging multiple binding entities, multispecific biotherapeutics have encountered more complex challenges than the conventional monospecific biotherapeutics during the process of drug discovery and development. Multispecific biotherapeutics often exhibit suboptimal physical and chemical properties, experience more difficult process development and optimization, and have faced more issues in pharmacokinetics, safety, and clinical development.

With more than 100 different formats available for format design [10], this abundant choice of selection also poses the challenge of picking the optimum format regarding the final product features, developability, manufacturability as well as freedom-to-operate or intellectual property. Whatever the final formats would be, the process of generating a candidate for multispecific biotherapeutics drugs would be complicated. New, efficient, and rapid methods for hit generation and candidate screening both in vitro and in vivo are highly desirable. For drug design, balancing affinity of individual arm to maximize therapeutic window, achieving synergism of multispecificity, and minimizing on-target and off-tumor toxicity are critical.

The complex structures of multispecific molecules also present significant challenges in achieving their drug-like properties [32], including favorable physical and chemical stability, solubility, viscosity, polyspecificity, immunogenicity, and pharmacokinetic properties. Compared to conventional monospecific antibody, much less is known about the developability properties of multispecific biotherapeutics. Continued improvements in expression and purification methods are needed for obtaining high yield in species of interest, generating minimal amount of unwanted species, and attaining simplicity in production methods [33]. Moreover, large experimental datasets for multispecific biotherapeutics, which are challenging and costly to produce, are needed for improving computational design and prediction.

Among the major classes of multispecific biotherapeutics, ADCs have the most commercialized drug products, ushering a burst of regulatory approvals in recent years. Yet most of clinical studies for ADCs remain unsatisfactory. Improvements on the potency of cytotoxic agents for lowering the minimum effective dose as well as on tumor selectivity for maximum tolerated dose are critical for the increase in therapeutic index. Producing more homogeneous and stable ADCs with control over their structural features are also important for the success of the next generation of ADCs. Mechanistic improvements for the potency of ADCs such as enhancing lysosomal delivery of ADC through engineering or co-targeting [221,222] should produce more innovative molecules. Beyond oncology, ADCs have opened up new therapeutic applications in the fields of infectious and inflammation diseases [223,224].

**Figure 2:**
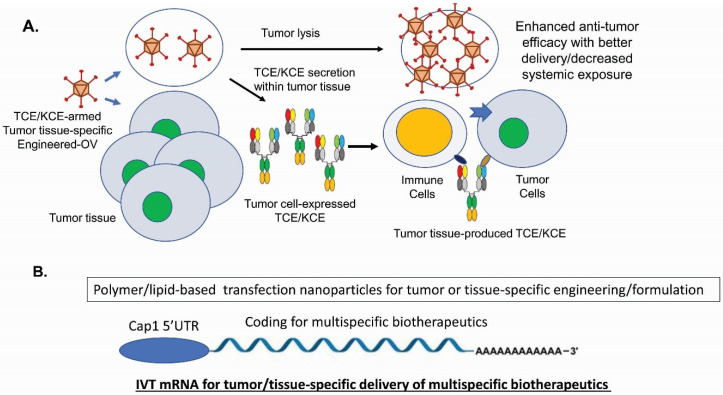
New technologies for multispecific biotherapeutics. (**A**). TCE and KCE-armed oncolytic viruses (OV) [227]. (**B**). In vitro transcribed (IVT) mRNA-encoded multispecific biotherapeutics [228].

For immune-cell engagers, there are new technologies on the horizon. Besides recent new designs on CiTE [136,137] and the costimulatory receptor for sustained T-cell proliferation [133,134], combining TCEs/KCEs with CAR-T/CAR-NK cells, iPSC-NK cells, checkpoint blockages or ADCs has opened up future investigations [5,6,225,226]. For cancer therapy, TCEs and KCEs-armed oncolytic viruses (OVs) have shown impressive efficacy in various tumor models [227] (Figure 2A).

With the recent FDA approval of T-VEC (Imlygic^TM^) for the treatment of advanced melanoma patients in the US in 2015 [229], OVs’ potential as potent anti-cancer biologics are established, despite the challenges such as antiviral immunity and insufficient delivery. TCEs- and KCEs-encoded OVs produce and secrete the multispecific drugs into tumor tissues so that endogenous T cells can be activated and directed to kill tumor cells and stromal cells for improved efficacy [27,230,231,232]. This combined strategy has significant advantages such as converting immunologically cold tumors into hot ones [233,234], minimizing on-target off-tumor toxicities [227,235], and enhancing T-cell infiltration into solid tumors [27]. To increase the therapeutic window of TCEs, high potency and low CRS toxicity can be achieved through next-generation protein engineering by decoupling tumor cell killing from cytokine release [23,236]. Moreover, multispecific biotherapeutics have issues of poor stabilities during long-term storage, aggregation tendency, presence of various impurities, as well as short serum half-life for non-IgG-like formats. To circumvent these manufacturing challenges and the limitations on tissue delivery, engineered mRNA-encoded [228] (Figure 2B) or DNA-encoded [237] multispecific molecules have shown good efficacy, safety profiles, and dosing advantages as well as potentials of specific target delivery. With the emerging and coming of age of many new technologies, we can foresee that multispecific biotherapeutics will play a bigger role in treating human diseases.

## Figures and Tables

**Table 3:** Approved products for multispecific biotherapeutics.

Approved Product Name	Year of Approval	Indication	Modality
catumaxomab (Removab^TM^)	2009 and withdrawal in 2017 for commercial reasons	Solid malignancies (malignant ascites owing to epithelial carcinomas)	bsTCE
blinatumomab (Blincyto^®^)	2014	Hematological malignancies [acute lymphoblastic leukemia (ALL) and B-ALL]	bsTCE
emicizumab (Hemlibra^®^)	2017	Routine prophylaxis of Hemophilia A with and without FVIII inhibitors	Matchmaker
moxetumomab pasudotox (Lumoxiti^TM^)	2018	Relapsed or refractory hairy cell leukemia	Tetherbody
gemtuzumab ozogamicin (Mylotarg^®^)	First-approved in 2000, withdrawal in 2010, and re-approved in 2017	Acute myeloid leukemia	ADC
brentuximab vedotin (Adcetris^®^)	2011	Hodgkin lymphoma, anaplastic large cell lymphoma, CD30-expressing mycosis fungoides	ADC
ado-trastuzumab emtansine (Kadcyla^®^)	2013	HER2+ metastatic breast cancer	ADC
inotuzumab ozogamicin (Besponsa^®^)	2017	Relapsed or refractory B-cell precursor acute lymphoblastic leukemia	ADC
enfortumab vedotin (Padcev^TM^)	2019	Locally advanced or metastatic urothelial cancer	ADC
Fam-trastuzumab deruxtecan-nxki (Enhertu^®^)	2019	HER2+ unresectable or metastatic breast cancer	ADC
Polatuzumab vedotin-piiq (Polivy^TM^)	2019	Relapsed or refractory diffuse large B-cell lymphoma	ADC
Belantamab mafodotin-blmf (Blenrep^TM^)	2020	Relapsed or refractory multiple myeloma	ADC
Sacituzumab govitecan (Trodelvy^TM^)	2020	Metastatic triple-negative breast cancer	ADC

**Table 4:** Target antigens, antibody, conjugation methods, linkers, payloads, and average drug antibody ratio (DAR) of approved antibody-drug-conjugates.

ADC Products	Target Antigen/Antibody	Conjugation Methods (Lys/Cys) Random/Site-Specific	Linker Payload	Average Drug Antibody Ratio
gemtuzumab ozogamicin (Pfizer)	CD33/humanized IgG4κ	Lys/random	*N*-acetyl-γ calicheamicin 1,2 dimethyl hydrazine dichloride	~1.5
brentuximab vedotin (Seattle Genetics)	CD30/chimeric IgG1	Interchain Cys/random	mc-vc-PABC-MMAE	~4
ado-trastuzumab emtansine (Roche)	HER2/humanized IgG1	Lys/random	SMCC-DM1	~3.5
inotuzumab ozogamicin (Pfizer)	CD22/humanized IgG4	Lys/random	*N*-acetyl-γ calicheamicin 1,2 dimethyl hydrazine dichloride	~5–7
enfortumab vedotin (Astellas)	Nectin-4/human IgG1κ	Interchain Cys/random	mc-vc-PABC-MMAE	~4
fam-trastuzumab deruxtecan-nxki (Daiichi Sankyo)	HER2/humanized IgG1κ	Interchain Cys/site-specific	mc-GGFG-DX-8951 derivative	~7.7
polatuzumab vedotin-piiq (Roche)	CD79b/humanized IgG1κ	Interchain Cys/random	mc-vc-PABC-MMAE	~4
belantamab mafodotin-blmf (GlaxoSmithKline)	BCMA/Afucosylated humanized IgG1	Interchain Cys/random	mc-MMAF	~4
sacituzumab govitecan (Immunomedics)	TROP2/humanized IgG1κ	Interchain Cys/random	Cl2A-SN38	~7.6
